# Evaluating the usability of public health data dashboards as information sources for professionals and the public: Findings from a case study with domain experts

**DOI:** 10.1111/hir.12532

**Published:** 2024-05-08

**Authors:** Bahareh Ansari

**Affiliations:** ^1^ Organization, Work, and Leadership Department, Queen's Management School Queen's University Belfast Belfast UK

**Keywords:** consumer health information, data visualisation, health literacy, human‐computer interaction, information literacy, patient education, public health

## Abstract

**Background:**

Recently, public health data dashboards have gained popularity as trusted, up‐to‐date sources of health information. However, their usability and usefulness may be limited.

**Objective:**

To identify the requirements of usable public health data dashboards through a case study with domain experts.

**Methods:**

Paired‐user virtual data collection sessions were conducted with 20 experts in three steps: (1) a monitored use of an existing dashboard to complete tasks and discuss the usability problems, (2) a survey rating user experience, and (3) an interview regarding the users and use cases. Data analysis included quantitative analysis of the survey findings and thematic analysis of the audio transcripts.

**Results:**

Analyses yielded several findings: (1) familiar charts with clear legends and labels should be used to focus users' attention on the content; (2) charts should be organized in a simple and consistent layout; (3) contextual information should be provided to help with interpretations; (4) data limitations should be clearly communicated; (5) guidance should be provided to lead user interactions.

**Discussion:**

The identified requirements guide health librarians and information professionals in evaluating public health data dashboards.

**Conclusion:**

Public health data dashboards should be designed based on users' needs to provide useful up‐to‐date information sources for health information consumers.

**Key Messages**

- Public health data dashboards are up‐to‐date, trusted information sources that can be used to fight misinformation and improve health literacy.
- Public health data dashboards should be designed based on their users' needs.
- Evaluation of public health data dashboards with domain experts identifies requirements for making data dashboards usable and useful sources of information.
- Information professionals can use the identified requirements to evaluate public health data dashboards as up‐to‐date, trusted information sources for health information consumers.

## INTRODUCTION

Disease surveillance data are a critical source of information for public health professionals to investigate the determinants of diseases and decide where resources are needed and what community‐level responses are required. These sources of information have recently become publicly available in a dashboard format (using interactive data visualisations) due to the increased availability of data and accessible visualisation software (Dasgupta & Kapadia, 2022). Data dashboards use visualisations (instead of lengthy reports with large tables of numbers) to make the surveillance data more accessible to a non‐technical audience, thereby enhancing data‐driven decision making among professionals and broadening their audience to the general public to encourage healthy lifestyles (Sarikaya et al., 2019). Fuelled by the COVID‐19 pandemic, data dashboards have become an integral part of public health efforts to share up‐to‐date data to inform the public about the pandemic status and fight the spread of online misinformation (Budd et al., 2020; Dixon et al., 2022).

Public health data dashboards are valuable resources for librarians and information professionals to share up‐to‐date disease statistics with professionals and the public (Ali & Gatiti, 2020). The value of data dashboards as up‐to‐date sources of information became more apparent during the COVID‐19 pandemic because of the urgent need for information and the quick spread of misinformation in the absence of quality information (Dixon et al., 2022), which could translate into accepting messages that discourage preventive behaviours such as vaccination (Love et al., 2020). During the COVID‐19 pandemic and beyond, health librarians and information professionals have been fighting the misinformation by fact‐checkers, myth‐busters, and guides to find and evaluate quality health information (Naeem & Bhatti, 2020). Teaching the skills to find, use, and evaluate data dashboards as trusted, up‐to‐date sources of health information can complement these efforts toward higher levels of health literacy in the population.

Public health data dashboards are traditionally used by two primary user groups: public health experts who solve routine and sometimes urgent tasks related to disease outbreaks and academics who do exploratory investigations and complex data analysis to identify disease patterns and risk factors (Perim & Lawonn, 2020). These user groups usually work in a specific area of public health, such as a specific disease. Thus, hereafter, they are referred to as domain experts, which characterize users with professional expertise about the specific disease area presented in the data dashboard. Although domain experts are the primary user group, the use of data dashboards for communication and education in recent years has expanded their users from experts to the general public (Sarikaya et al., 2019).

Despite the increased availability of public health data dashboards, evaluation with users has been limited, resulting in usability problems in existing tools, which limited their usefulness. For example, a study of John Hopkins and WHO COVID‐19 dashboards shows that these dashboards may leave visitors with anxiety and information overload instead of helping with informed decision making (Çay et al., 2020). Another study of COVID‐19 data dashboards shows they violate the usability principle and have limited usefulness for their intended users (Monkman et al., 2021). Evaluating these tools by their users could establish requirements that guide health librarians and information professionals to evaluate the usability of a data dashboard as a source of information for health information consumers.

The current study investigated the usability of public health data dashboards through a case study with domain experts in sexually transmitted infections (STIs). STIs are among the most common infections in the United States (Kreisel et al., 2021), with substantial healthcare‐related costs (Chesson et al., 2021). Given the scope of the STI epidemic, this case study can provide valuable insights into evaluating public health data dashboards for similar public health focus areas. Moreover, online sources of health information about STIs were associated with higher knowledge levels about their consequences (Ansari, 2021), which makes them useful case studies for public health practice. Domain experts were chosen for evaluation because, based on the author's experience developing a data dashboard for the New York State Department of Health, they were the most frequent users of STI data dashboards (Ansari, 2022; Ansari & Martin, 2024). Moreover, they were suitable for usability testing because, although knowledgeable about STIs, most had little experience with data dashboards. Therefore, they were not expected to understand data dashboards better than a layperson.

## METHODS

### Overview

Virtual meetings were conducted with domain experts to examine the usability problems of an existing data dashboard and discuss general users and use cases of public health data dashboards. Meetings were designed and moderated by the author. Each meeting had three steps: monitored paired‐user activity, usability and user experience survey, and an exit interview. Quantitative data were collected from responses to the survey, and qualitative data were collected from the audio transcripts. Summary statistics were used to analyse the survey results, and an inductive thematic analysis method was used to analyse the transcripts (Braun & Clarke, 2006). The author's university institutional review board reviewed and approved data collection and analysis methods.

### Case study: Data dashboard of STIs in Arizona

The data dashboard of STIs in Arizona was used for data collection. This dashboard was selected after the authors' prior work (Ansari & Martin, 2022) examining all the STI data dashboards available on the state health department websites in the United States (*N* = 13 as of June 1, 2021). The Arizona STI data dashboard was selected because it had many opportunities to explore, including various visualisations with different interactive features and granular data (i.e., diagnoses broken down by demographic information).

**FIGURE 1:**
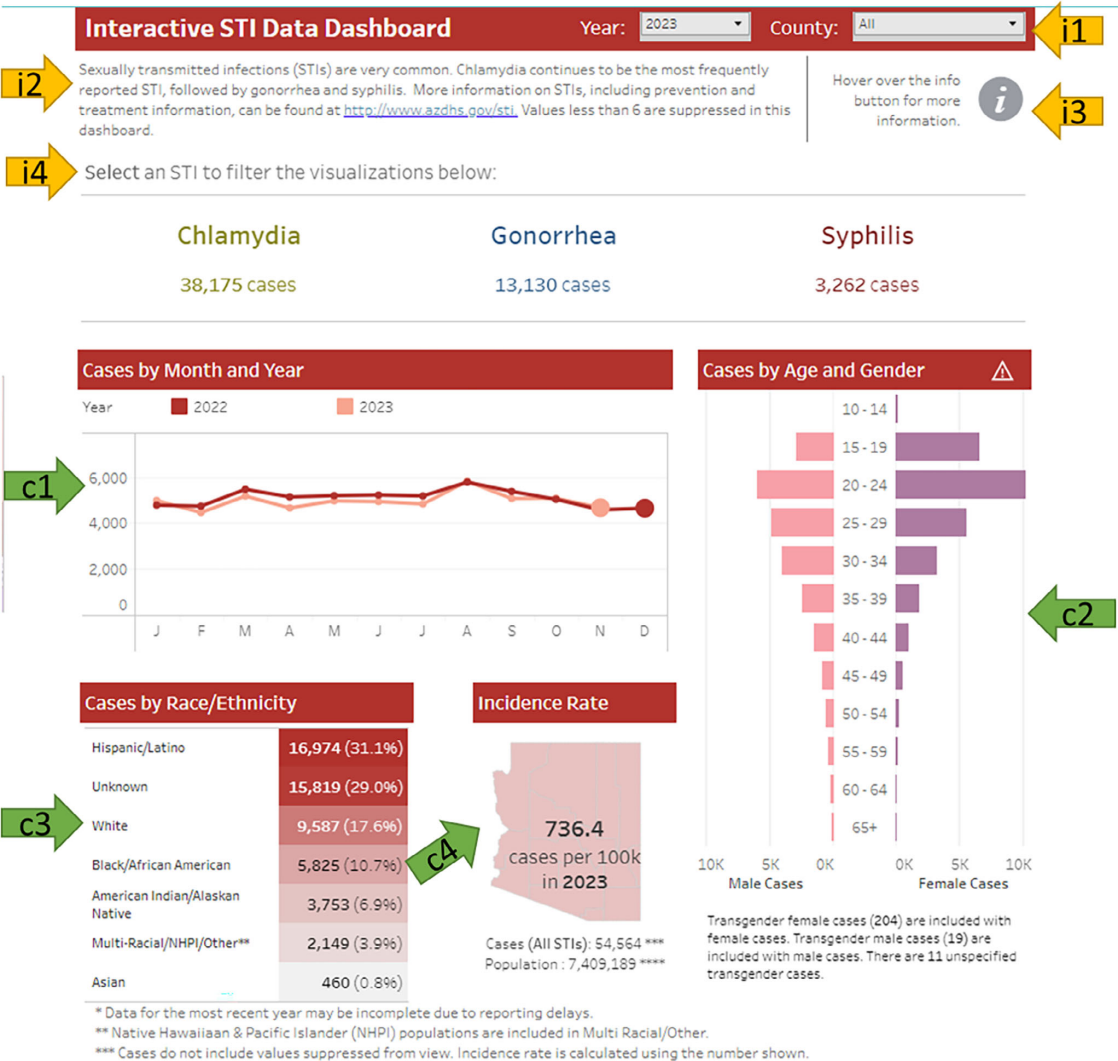
Screenshot of the homepage of the examined public health data dashboard. [Colour figure can be viewed at wileyonlinelibrary.com]

Figure 1 is a screenshot of the examined data dashboard's homepage. The four green arrows point to the four charts: arrow ‘c1’ points to a line chart showing the breakdown of the reported cases by month and year, arrow ‘c2’ points to a diverging bar chart showing the breakdown by age and gender, arrow ‘c3’ points to a highlight table showing the breakdown by race and ethnicity, and arrow ‘c4’ points to a polygon that shows the overall incidence rate in the state of Arizona. The yellow arrows point to the interactive features: arrow ‘i1’ points to two filters for selecting a year and county, arrow ‘i2’ points to a note including an external link to the main website for information on treatment and prevention, arrow ‘i3’ points to an interactive button (with ‘i’ icon) for more information, and arrow ‘i4’ points to a filter for selection of a specific infection (i.e., chlamydia, gonorrhoea, or syphilis).

**FIGURE 2:**
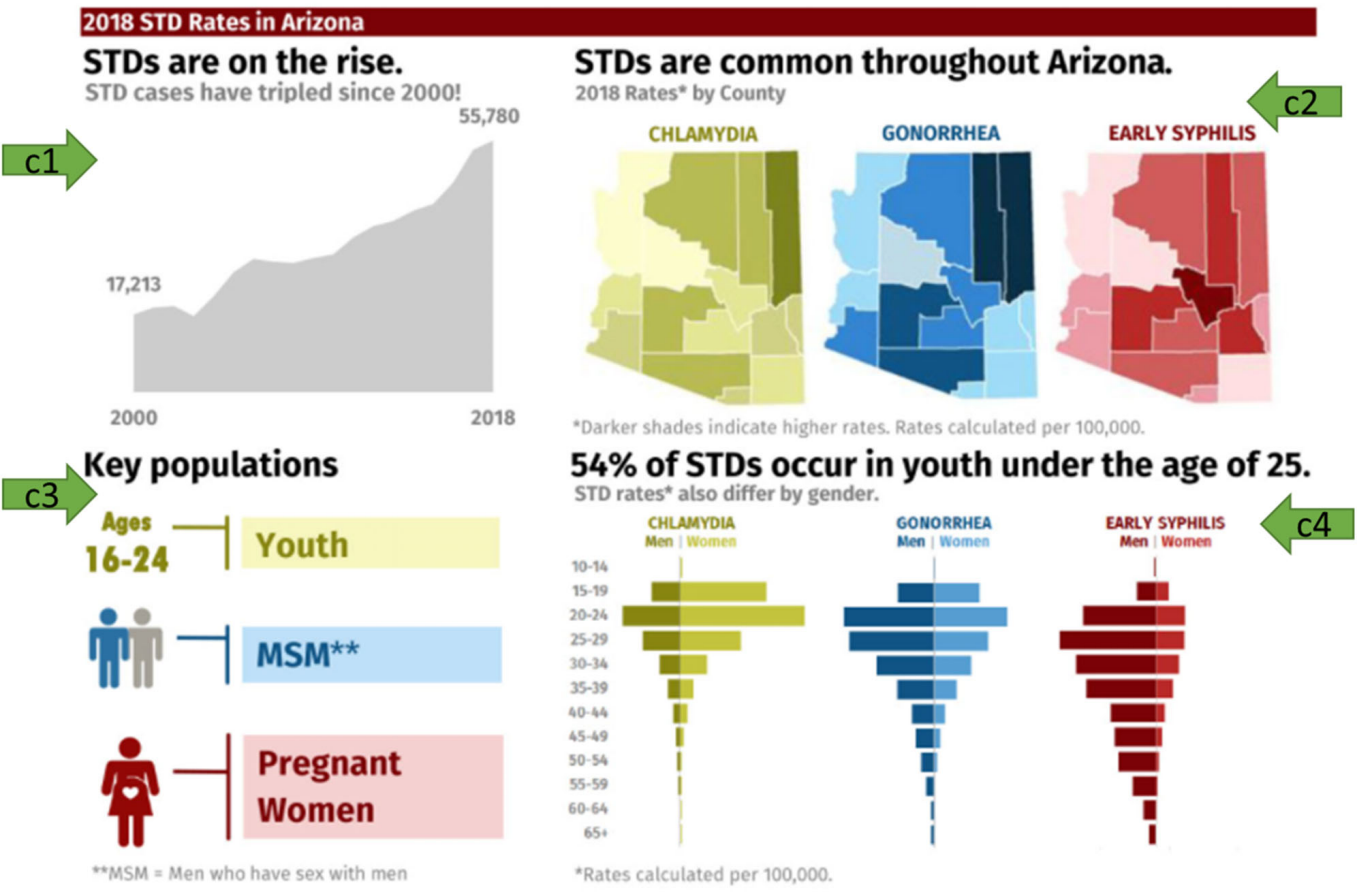
Screenshot of one of the pages on the examined public health data dashboard. Date of the screenshot: October 30, 2021. [Colour figure can be viewed at wileyonlinelibrary.com]

Figure 2 is a screenshot of one of the pages that opened on‐demand from the homepage. The four green arrows in Figure 2 point to the four charts on this page: arrow ‘c1’ points to an area chart showing the trend from 2000 to 2018, arrow ‘c2’ points to a faceted map depicting the distribution of the three infections throughout the state, arrow ‘c3’ points to a pictogram demonstrating the key populations, and arrow ‘c4’ points to a faceted diverging bar chart showing the distribution of the three infections among males and females in different age groups.

### Participant recruitment

Participants were recruited from domain experts working in the field of STIs in New York State. These experts were targeted for recruitment because they were similar to the primary end‐users of the examined case study (the Arizona STI data dashboard) and were accessible to the author through existing relationships. Recruitment was done with the help of the New York State Department of Health AIDS Institute, whose portfolio includes HIV, STIs, viral hepatitis, and drug user health. An email was sent to the HIV Advisory Board (a group of volunteers who provide feedback on the public health programmes designed by the New York State Department of Health) and a listserv for community‐based organizations that deliver public health services related to HIV and STIs. The recruitment email included a flyer introducing the research project and a registration link that guided participants to registration. After the registration, participants received another email to schedule the virtual data collection sessions. The registration link was open throughout the data collection period, registering 28 persons, of which 20 were scheduled for virtual data collection. The registration was stopped when saturation was observed in new concepts that emerged from subsequent sessions.

### Data collection

Data collection sessions were conducted virtually on Zoom (during July and August 2021). Each data collection session took 2 h and included two participants. At the beginning of each meeting, the moderator explained the purpose of the study, the data collection and analysis procedure, and the methods to protect participants' data. The participants were allowed to ask their questions and were then asked to give informed consent to continue the meeting. Data were collected in three steps that are described below. Table 1 provides more details about the questions that were asked and the data collected in each step.

**TABLE 1:** Data collection steps.

Task/goal	Question
Step 1. Monitored paired‐user activity (collected data: researcher's observations and audio transcripts)	
Exploration	How many new cases of gonorrhoea were diagnosed in December 2020? And how did this number change compared to December 2019? (Follow up: what problems did you encounter when answering this question?)
Association	Does the risk of acquiring gonorrhoea go up or down with age? (Follow up: what problems did you encounter when answering this question?)
Pattern identification	Is there any geographical pattern to the gonorrhoea rate in Arizona? (Follow up: what problems did you encounter when answering this question?)
Comparison	What racial/ethnic groups have the highest number of cases? What racial/ethnic groups have the highest rates? (Follow up: what problems did you encounter when answering this question?)
Verification	Can you find any explanation for potential problems of gender classification in this dashboard? (Follow up: what problems did you encounter when answering this question?)
Policy development	Can you recommend a population group for increased screening or a region for funding allocation? (Please only use the information provided on this dashboard and not your prior knowledge) (Follow up: what problems did you encounter when answering this question?)
Dissemination	Suppose that you are helping with an awareness campaign for gonorrhoea in Arizona. Identify the most appropriate chart or charts for public dissemination. What are possible methods for sharing? Try one of the methods you identified for sharing and discuss among yourselves if this method would be efficient in public dissemination.
Step 2. Usability and user experience survey (collected data: quantitative scores)	
Satisfaction	The dashboard was overall: Boring 1 2 3 4 5 6 7 Satisfying
Enjoyment	The dashboard was overall: Unpleasant 1 2 3 4 5 6 7 Enjoyable
Fun	The dashboard was overall: Annoying 1 2 3 4 5 6 7 Fun
Motivating	The dashboard was overall: Gimmicky 1 2 3 4 5 6 7 Motivating
Provocative	The dashboard was overall: Childish 1 2 3 4 5 6 7 Provocative
Engagement	The dashboard was overall: Frustrating 1 2 3 4 5 6 7 Engaging
Challenge	The dashboard was overall: Make you feel guilty 1 2 3 4 5 6 7 Challenging
Surprise	The dashboard was overall: Make you feel stupid 1 2 3 4 5 6 7 Surprising
Effectiveness	The dashboard allowed me to access the information I needed and complete the tasks that I wanted. Strongly disagree 1 2 3 4 5 6 7 Strongly agree
Efficiency	Once I learned how to use the dashboard, I was able to carry out the tasks quickly. Strongly disagree 1 2 3 4 5 6 7 Strongly agree
Safety	The dashboard design prevented me from making serious errors, and it was easy to recover from small errors. Strongly disagree 1 2 3 4 5 6 7 Strongly agree
Utility	I was able to carry out the tasks the way I wanted. Strongly disagree 1 2 3 4 5 6 7 Strongly agree
Learnability	I was able to quickly learn how to use the dashboard. Strongly disagree 1 2 3 4 5 6 7 Strongly agree
Memorability	It will be easy to remember how to use this dashboard if I need it in the future. Strongly disagree 1 2 3 4 5 6 7 Strongly agree
Step 3. Exit interview (collected data: audio transcripts)	
Overall reaction	Overall, how did you feel about the capabilities provided by the dashboard? Can you point to the source of your feeling (What exactly made you interested or bored)?
Important requirements	What were the least and most desirable features on the Arizona dashboard?
Potential users	Who do you think would (should) use the public health data dashboard?
Potential use cases	What would these users want to do with the dashboard? Would it be something similar or different than the questions you answered today?
Additional requirements	What additional capabilities should a public health data dashboard provide?

In the first step, a monitored paired‐user activity was conducted, where the participants were asked to work together to complete seven tasks using the selected dashboard. Monitored user activity was chosen because it is an established method for finding usability problems (Sharp et al., 2019). Using this method, usability problems emerge by observing users' regular work with the technology (Sharp et al., 2019). In addition, while working on the tasks, the moderator asked users to reflect on the interface design and the problems they encountered. The seven tasks included exploration, pattern identification, association, comparison, verification, policy development, and dissemination, designed based on common tasks in public health visual analytics (Perim & Lawonn, 2020).

In the second step, the participants were asked to complete a survey about their user experience. The survey was designed based on established dimensions of usability and user experience. For example, a product is usable if it is easy to learn, memorable, and safe to use, and it enables users to carry out their activities effectively and efficiently (Sharp et al., 2019). Therefore, usability questions measured different dimensions of usability, including safety, memorability, learnability, effectiveness, efficiency, and utility. Moreover, common positive user experiences are engagement, enjoyment, satisfaction, surprise, fun, challenge, motivation, and provocative, which were asked in comparison to their opposite experiences, such as boring, unpleasant, patronizing, and annoying (Sharp et al., 2019).

In the third step, the participants were asked to reflect on their overall experience in an exit interview. Furthermore, the participants were asked about the least and most desirable features they saw on the dashboard and their opinions about the potential users and use cases of data dashboards.

### Data analysis

Survey responses were analysed using descriptive statistics and the Kruskal–Wallis test to test statistical differences between groups. Kruskal–Wallis was chosen because it is a non‐parametric test for analysis of the variances (equivalent to ANOVA) that do not make any assumption about the underlying distributions, making it suitable for the small sample size and unbalanced groups of this study (Corder & Foreman, 2009). Audio transcripts were automatically developed by the Zoom software. Transcription errors were corrected manually by comparing the original recordings with the transcripts. Inductive thematic analysis was conducted to identify the themes that emerged from the data and focused on the usability problems instead of developing a conceptual framework (Braun & Clarke, 2006). The coding process had three steps. First, a careful reading of all transcripts identified meaningful units of text. Second, each unit was categorized into one or more codes that best described the content. These codes were inductively derived from the data and were not based on an a priori coding scheme. Third, to ensure the reliability of the codes, a second coder independently assigned codes to 2 of the 10 meeting transcripts. We checked our coding consistency, discussed disagreements, and revised the codes to ensure that different coders could consistently use the codes.

The inductive thematic analysis resulted in 41 codes grouped into five categories. Table 2 displays a complete list of the codes and categories. The first category captured the usability problems related to chart reading, including confusing colour codes, insufficient legends and labels, and unfamiliarity (with new chart types, such as diverging bar charts). The second category captured the usability problems related to data limitations, including missing data, insufficient granularity, insufficient contextual information, presenting data limited to some STIs, and outdated data. The third category captured the usability problems related to information organization, including busy layout, inconsistency (between the pages), and the overall structure of the information organization. The fourth category was the usability problems related to contextual information, including illegible text, unjustified conclusion(s), user misinterpretation, and jargon without explanation. The last category was the usability problems related to interactivity, including hidden user input boxes, unclear feedback upon interaction, and non‐interactive charts. The usability problems that did not occur repetitively throughout the study were coded as other problems.

**TABLE 2:** Codes used in qualitative coding.

Categories	Codes
Usability problems	Chart reading Confusing colour codes Insufficient legends and labels Unfamiliarity Data limitations Missing data Insufficient granularity Limited to some STIs Outdated data Information organization Busy layout Inconsistency Contextual information Illegible text Unjustified conclusion User misinterpretation Jargon without explanation Interactivity problems Hidden input boxes Unclear feedback upon interaction Non‐interactive charts Other problems
Suggestions	Chart reading Minimize the use of red colour Prefer bar charts and line charts Data limitation Provide granular data Make data tables available Visualize more variables Provide different measures (numbers and rates) Information organization Add guidance for navigation Contextual information Explain screening vs. incidence Add narratives of impacts, successes, and treatment Explain data classifications and limitations Interactivity problems Add interactive features (filters, sliders, pop‐up boxes) Other suggestions
Users	Practitioners Academics Others (Journalists, advocates, etc.)
Use cases	Overview Investigate impacted populations Download data for further analysis

## RESULTS

### Participants

Table 3 provides information on the participants. Participants represented domain experts in the STI field in New York State. Among the 20 participants, 10 identified as cisgender women, seven as cisgender men, one as transgender man, one as transgender woman, and one as non‐binary. All participants had some college education or higher; 15 out of 20 participants were 30–49 years old, two were 18–29, and three were 50–69. In terms of prior familiarity with data dashboards, participants were classified into five levels of familiarity: (1) never seen data dashboards before, (2) had seen screenshots of data dashboards in settings outside of work only (e.g., news), (3) have seen screenshots of data dashboards in meetings and seminars, (4) have used data dashboards, to read and learn about statistics of a disease or other things (e.g., election results), (5) have used data dashboards, and interacted with them to make customized data visualisations for personal or professional use. Thirteen out of 20 participants have used data dashboards (levels 4 and 5), six participants had only seen but never used dashboards before (levels 2 and 3), and one participant had never seen any data visualisation tool (level 1).

**TABLE 3:** Participants' characteristics.

Participant^a^	Current professional role	Highest education achieved	Age group (years)	Level of familiarity with data dashboards^b^
Participant 1–1	Direct service provider	Some college or associate degree	50–69	Level 3
Participant 1–2	Volunteer	Graduate degree	30–49	Level 3
Participant 2–1	Direct service provider	Graduate degree	30–49	Level 5
Participant 2–2	Direct service provider	Some college or associate degree	50–69	Level 1
Participant 3–1	Healthcare administration	Bachelor's degree	30–49	Level 4
Participant 3–2	Healthcare administration	Bachelor's degree	30–49	Level 4
Participant 4–1	Healthcare administration	Bachelor's degree	18–29	Level 5
Participant 4–2	Direct service provider	Graduate degree	30–49	Level 5
Participant 5–1	Direct service provider	Graduate degree	50–69	Level 4
Participant 5–2	Healthcare administration	Graduate degree	30–49	Level 4
Participant 6–1	Direct service provider	Bachelor's degree	18–29	Level 3
Participant 6–2	Direct service provider	Graduate degree	30–49	Level 4
Participant 7–1	Healthcare administration	Some college or associate degree	30–49	Level 2
Participant 7–2	Direct service provider	Some college or associate degree	30–49	Level 4
Participant 8–1	Healthcare administration	Bachelor's degree	30–49	Level 4
Participant 8–2	Healthcare administration	Bachelor's degree	30–49	Level 3
Participant 9–1	Healthcare administration	Graduate degree	30–49	Level 4
Participant 9–2	Healthcare administration	Bachelor's degree	30–49	Level 4
Participant 10–1	Healthcare administration	Graduate degree	30–49	Level 4
Participant 10–2	Direct service provider	Bachelor's degree	30–49	Level 2

*Note*: Gender identity is not included in this table to ensure anonymity.

^a^ There were 10 sessions and two participants in each session. The name coding has two parts to distinguish the two participants in each session: the initial one or two digits to refer to the meeting number (1–10) and the last digit (1, 2) to refer to the participant number. For example, Participant 1–1 and Participant 1–2 were present in meeting #1, and Participant 10–1 and Participant 10–2 were present in meeting #10.

^b^ There were five levels of familiarity with data dashboards: (1) never seen data dashboards before, (2) have seen screenshots of data dashboards in settings outside of work only (e.g., news), (3) have seen screenshots of data dashboards in meetings and seminars, (4) have used data dashboards, to read and learn about statistics of a disease or other things (e.g., election results), (5) have used data dashboards, and interacted with them to make customized data visualisations for personal or professional use.

### Survey findings

**FIGURE 3:**
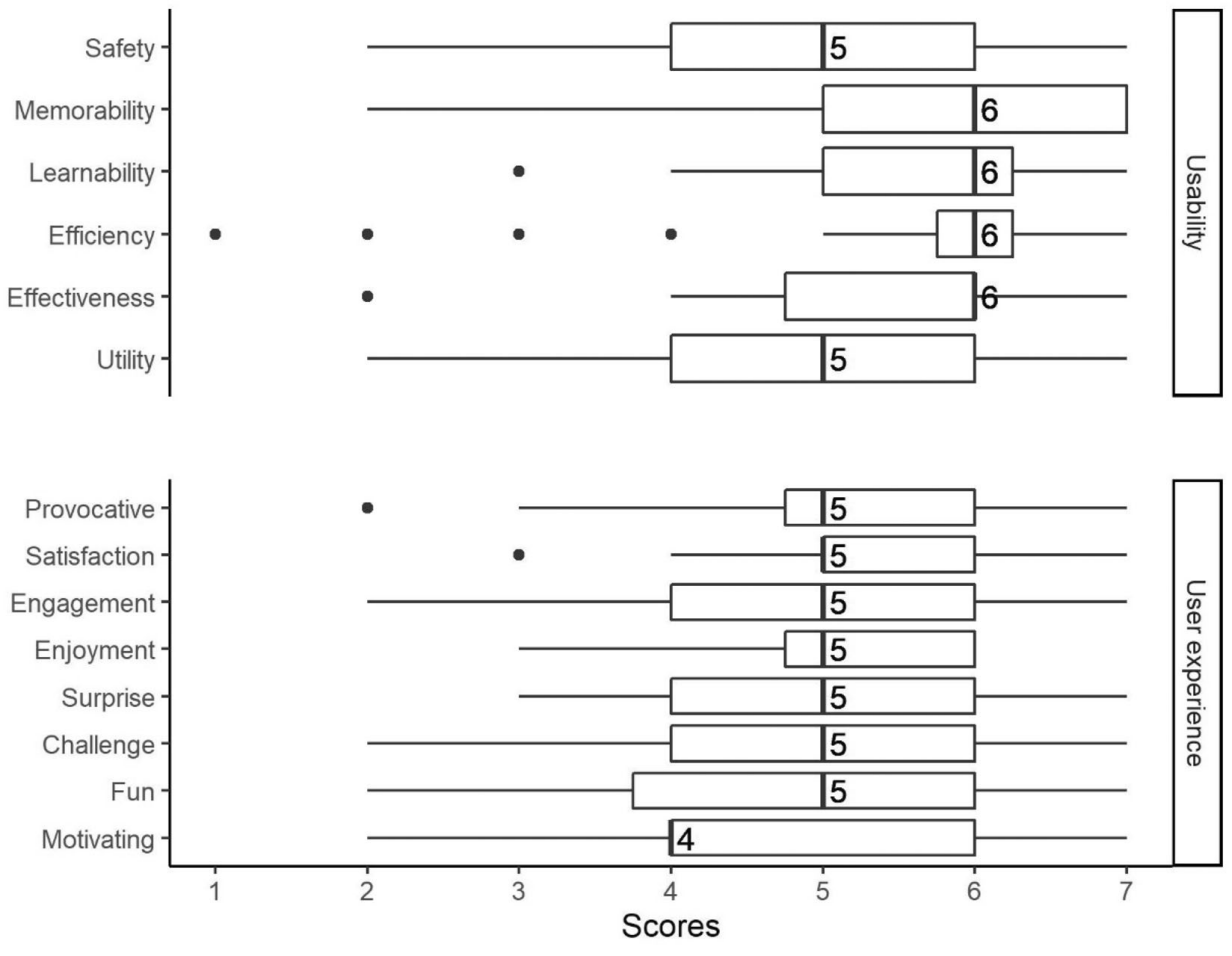
Box plots of the scores given to each of the usability or user experience goals. The box plot summarizes the 20 participants' scores for each usability and user experience item on a 7‐point scale. The numbers on the chart are labels for medians. Some boxes appear to be one‐sided. For example, the median and Q1 were the same values for the motivating experience, which means there was no variation in the lower part of the data (5 people gave a score of 4). Also, for efficiency, most participants agreed, except for four outliers.

Figure 3 shows the distribution of survey responses. Participants' answers showed that they generally perceived an acceptable level of usability on different dimensions, including effectiveness (median = 6 on a 7‐point Likert scale), efficiency (median = 6), learnability (median = 6), memorability (median = 6), safety (median = 5), and utility (median = 5). Furthermore, responses to the user experience questions suggest an overall positive experience in different dimensions of emotional response, including challenge (median = 5 on a 7‐point Likert scale), engagement (median = 5), enjoyment (median = 5), fun (median = 5), motivating (median = 4), provocative (median = 5), satisfaction (median = 5), and surprise (median = 5).

Tables 4 and 5 show the survey responses by participant groups. Table 4 shows the median and interquartile range (IQR) of usability scores, and Table 5 shows the median and IQR of user experience scores. For example, participants in the age group 18–29 gave a median score of 3.5 (IQR = 1.5) to the safety dimension of the usability (in response to ‘the dashboard design prevented me from making serious errors, and it was easy to recover from small errors’.). The median score of 3.5 on a 7‐point Likert scale means that, on average, this group was neutral about the dashboard's ability to prevent errors (with scores between 3.5 (±1.5) or between 2 and 5). Similarly, participants in the age group 18–29 gave a median score of 5 (IQR = 1) to the satisfying dimension of user experience (in the selection of 1 to 7 from boring to satisfying). The median score of 5 means that, on average, this group rated their experience of using the dashboard closer to satisfying and farther from boring.

**TABLE 4:** Usability scores by different participant groups.

Characteristic	Sample size	Safety	Memorability	Learnability	Efficiency	Effectiveness	Utility
Age group							
18–29	2	3.5 (1.5)	4.5 (2.5)	5 (2)	3.5 (2.5)	4 (2)	4 (1)
30–49	3	5 (2)	6 (2)	6 (1.5)	6 (0.5)	5 (1.5)	5 (2)
50–69	15	4.5 (0.5)	7 (0.5)	6 (0)	6 (1)	6 (0.5)	6 (1)
*p*‐value		0.65	0.33	0.09	0.23	0.40	0.50
Education							
Associate degree	4	5 (1.5)	6.5 (1.25)	6 (0.5)	6.5 (1.25)	6.5 (1)	6.5 (1.5)
Bachelor's degree	8	5 (2.5)	6 (1.5)	6 (1.5)	6 (1)	6 (1.25)	5 (2.25)
Graduate degree	8	4.5 (1.25)	6 (2)	6 (1)	6 (0.5)	5 (1.25)	5 (1.25)
*p*‐value		0.56	0.96	0.82	0.35	0.08	0.33
Level of familiarity							
Level 1	1	5 (0)	7 (0)	6 (0)	7 (0)	6 (0)	6 (0)
Level 2	2	5 (1)	5.5 (0.5)	5.5 (0.5)	6 (0)	6 (0)	5 (1)
Level 3	4	4.5 (0.5)	6 (0.75)	6 (0.75)	5.5 (1.5)	6 (0.75)	5.5 (1.75)
Level 4	10	5 (1.75)	7 (1)	6 (1)	6 (1)	5.5 (1)	5 (0.75)
Level 5	3	2 (2)	5 (1.5)	5 (1)	2 (1.5)	2 (1)	3 (1)
*p*‐value		0.22	0.37	0.25	0.15	0.07	0.46

*Note*: The numbers in the table represent each participant group's median (interquartile range) based on their characteristic. The *p*‐values are the result of the Kruskal–Wallis test for testing the probability of significant differences between the participant groups' responses.

**TABLE 5:** User experience scores by different participant groups.

Characteristic	Sample size	Satisfaction	Provocative	Engagement	Enjoyment	Surprise	Challenge	Fun	Motivating
Age group									
18–29	2	5 (1)	5 (1)	3 (1)	5 (1)	4 (0)	6 (1)	4.5 (1.5)	5 (1)
30–49	3	5 (0.5)	5 (1.5)	5 (2)	5 (0.5)	5 (2)	4 (1)	5 (2)	4 (2)
50–69	15	5 (0.5)	6 (0.5)	6 (0)	6 (0)	5 (0.5)	6 (1)	5 (1.5)	6 (1.5)
*p*‐value		0.63	0.71	0.07	0.32	0.21	0.23	0.99	0.88
Education									
Associate degree	4	5.5 (1.25)	5.5 (1.25)	6 (1)	6 (0.25)	5.5 (1.25)	6 (0.5)	5.5 (1.5)	5.5 (3)
Bachelor's degree	8	5 (1.25)	5 (1.5)	4.5 (1.25)	5 (1)	4.5 (1)	4.5 (1.5)	4.5 (3)	4 (1.5)
Graduate degree	8	5 (0.25)	5 (1.5)	5.5 (1.25)	5 (0.5)	4.5 (2.25)	4.5 (1)	5 (0.5)	5 (2)
*p*‐value		0.42	0.71	0.97	0.58	0.17	0.34	0.5	0.13
Level of familiarity									
Level 1	1	5 (0)	6 (0)	6 (0)	6 (0)	5 (0)	6 (0)	6 (0)	7 (0)
Level 2	2	5 (0)	5 (0)	4 (1)	5 (0)	5 (1)	4 (0)	5 (0)	4 (0)
Level 3	4	6 (0.5)	5.5 (1.5)	5 (2.25)	6 (0.75)	4.5 (1.25)	5.5 (1)	4.5 (3.25)	5 (2.5)
Level 4	10	5 (0.75)	5 (1.75)	6 (1)	5 (0)	5 (1.5)	4.5 (1)	5 (1.75)	4.5 (2.5)
Level 5	3	4 (1)	4 (1)	3 (1.5)	4 (0.5)	4 (1.5)	4 (2)	3 (1)	4 (1)
*p*‐value		0.44	0.27	0.68	0.08	0.64	0.22	0.87	0.78

*Note*: The numbers in the table represent each participant group's median (interquartile range) based on their characteristic. The *p*‐values are the result of the Kruskal–Wallis test for testing the probability that there are significant differences between the participant groups' responses.

Overall, Tables 4 and 5 show little differences between participant groups except for two groups with lower usability and user experience scores: those 18–29 years old and those with level 5 familiarity with data dashboards (e.g., prior extensive experience with data dashboards). This might be because these groups generally had higher expectations from data dashboards or discovered more usability problems because they explored more functions on the evaluated data dashboard. However, these differences were not statistically significant (shown with high *p*‐values), and the sample sizes of these groups were small.

### Qualitative analysis findings

Analysis of the audio transcripts yielded some themes regarding the usability problems and possible solutions in several areas, including layout, charts, data, textual explanation, and interactivity. Table 6 summarizes the problems and suggestions for improvement elicited by participants. Themes are described below, with quotes from data for illustration.

**TABLE 6:** Usability problems and suggestions for improvement elicited by participants.

Categories of usability problems	Specific usability problems	Participants' suggestions for improvement
Chart reading	Confusing colour codes Insufficient legends and labels Unfamiliarity	Minimize the use of red colour Prefer bar charts and line charts
Data limitations	Missing data Insufficient granularity Limited to some STIs Outdated data	Provide granular data Make data tables available Visualize more variables Provide different measures (numbers and rates)
Information organization	Busy layout Inconsistency between pages	Add guidance for navigation
Contextual information for interpretations	Jargon without explanation Not using inclusive language Illegible text Unjustified conclusion User misinterpretation	Explain screening vs. incidence Add narratives of impacts, successes, and treatment options Explain data classifications and limitations
Interactivity problems	Hidden input boxes Unclear feedback upon interaction Not interactive charts	Add interactive features (filters, sliders, pop‐up boxes)

The first set of usability problems was related to chart reading. The most common usability problems with reading charts were insufficient legends and labels and confusing colour codes. Confusing colour codes were experienced in two cases. First, the use of the same colour to represent different concepts was confusing to participants, as illustrated in the following quote from Participant 4–2: ‘I think they just put a lot of information here that is disconnected from each other, but they are colour‐coded in the same way. So, it seems that it is related, where it is not’. Second, the inverse use of stereotypical colours to distinguish males vs. females was confusing to participants, as illustrated by the following quote from Participant 5–2 about arrow ‘c2’ on Figure 1: ‘I keep looking at pink and thinking that it is the female cases. Because we are dealing with pink and purple here, I think that is confusing. I am not suggesting that they do like pink and blue, but, I do not know, maybe purple and green, or something that's not so distinctly identified to women’.

The second set of usability problems was related to information organization, particularly the difficulty of finding some information elements, illustrated by the following quote from Participant 5–2: ‘On the top, you have to click into each one of the different sections to get like chlamydia versus gonorrhoea versus syphilis. So, it does not make sense to me, then to have to go down to the second section and choose chlamydia again. I feel like if you are clicking on chlamydia, give me all the chlamydia information, do not make me search for it just like, put it all together’. Less common information organization problems were busy layout (i.e., many charts on one page) and inconsistency, which led to difficulties finding pieces of information (e.g., one section had information from 2019 to 2021, and the other had information up to 2018).

The third set of usability problems was related to data limitations. The most common problems included missing data, insufficient granularity, and outdated data. Participants were concerned to see a large proportion of cases with missing information about race and ethnicity, as illustrated by the following quote from Participant 3–1 about arrow ‘c3’ on Figure 1: ‘Unknown race/ethnicity really threw me off. Because hardly ever I see a chart that unknown is so high. It just lets me know that their questionnaires for whomever they are gathering this data are not precise or deep as they should be’. Participants' concerns about insufficient granularity pertained to two cases: geographical information (e.g., data by county or zip code) and categorical data (e.g., diverse gender, race, and ethnicity classification), both of which could limit the usefulness of the data or the fairness of the provided services to the community that most need it. As an example of these concerns, the following quote illustrates Participant 5‐1's concern about the insufficient granularity of the geographic information: ‘Looking at the counties on the map, you cannot see where urban centres are vs. rural areas. There is probably a county seat in every one of them, where the population is denser, so maybe that is where the clusters are. With the map of counties, you really do not know about the dispersion of the population’.

The fourth set of usability problems was related to the lack of contextual information to understand the measures and insights. The most common usability problems were the insufficient explanation about disease maps that might lead to user misinterpretation, illustrated by the following quote from Participant 8–1: ‘The only thing you got to be careful, in my opinion, with disease maps, is to not feed into stigma as well so like if consumers are looking at this, they may be like, Oh, well I want to avoid this area because they have this [disease], and I want to go somewhere else’. Less common usability problems with contextual information included jargon without explanation, illegible text, and unjustified conclusions (i.e., conclusions not supported by the presented charts).

The fifth set of usability problems was related to interactivity that dealt with the different opportunities users had to interact with the charts to customize the views according to their needs. The interactivity features on the examined dashboard were filtering, hover effect (pop‐up messaging), and show or hide views. The most common interactivity problems on the examined dashboard were the hidden input boxes (element ‘i1’ in Figure 1) and unclear feedback upon interaction (element ‘i4’ in Figure 1), leading to participants' confusion. The following quote from Participant 6–2 illustrates the interactivity problems with element ‘i4’ in Figure 1: ‘When you click on something, it gives you the same graphs. But how was I supposed to know that it would be any different? Graphs do not change. It's the same graphs; we go over here, it is the same graphs. So now I'm like, okay, I do not see the difference on the homepage that's supposed to be for all three. I wouldn't know that unless I played with it’. A less common usability problem was the need for more interactivity (especially pop‐up messaging to give more details) on the static charts.

### Participant differences

Some themes were exclusive to some participants. For example, only participants with level 4 and 5 data dashboard familiarity (i.e., those who used data dashboards before, whether to read charts or make customized data visualisations) discussed the need for more interactivity on the charts. Furthermore, suggestions were given mainly by participants with levels 4 and 5 of data dashboard familiarity. On the other hand, most usability problems (including insufficient granularity, insufficient legends and labels, and not using inclusive language) were consistently reported by participants of all levels.

The observed participants' differences in the qualitative data are supported by the differences in the quantitative data from survey responses (Tables 4 and 5). Both data sources suggest few differences between participants in the usability problems they encountered and their overall experience. Moreover, both data sources suggest that those more familiar with data dashboards experienced more usability problems (especially about the interactive features), which could be attributed to their higher expectations or more explorations of the evaluated data dashboard.

### Exit interview findings

In the exit interviews, participants identified three main user groups with diverging use cases and requirements: (1) practitioners (e.g., clinicians, service providers, outreach workers) who are interested in the investigation of impacted populations for evidence‐based practice and funding applications; (2) academics, who are interested in detailed data analysis; (3) others (e.g., advocacy groups, journalists, students), who are interested in an overview of trends and disparities for educational and advocacy purposes. The identified user groups have diverging requirements. The requirements for public health practitioners include more interactive features and opportunities for exploration and finding gaps. The requirements for academic users include downloading the dataset for linking with other databases and advancing research. Finally, the requirements for other users are a simple presentation of findings with textual explanations. Furthermore, participants' answers to the least and most desirable features determined that some usability problems were more critical for participants, including insufficient labelling on the charts, missing data, the possibility of user misinterpretation, and unclear feedback upon interaction.

## DISCUSSION

Through monitoring the use of a data dashboard and a follow‐up survey and interview with domain experts, the current study found some requirements for a usable public health data dashboard that can guide information professionals in evaluating these information sources. The significance of these requirements, in relation to the wider literature, is discussed in the following paragraphs. Moreover, this study found that diverse user groups use public health dashboards and have diverging needs. More interactivity is needed for experts, and more narratives are needed for novice users, requiring public health data dashboards to have flexible features that can be skipped by one group while used by the other. Alternatively, some data dashboards might provide different versions that address the needs of different user groups (Dasgupta & Kapadia, 2022; Shneiderman, 2000).

This study found that familiar charts (with clear colour codes, legends, and labels) and simple, consistent layouts should be used to focus users' attention on the content rather than the visualisation technique. Moreover, some charts (especially maps) need contextual information to help with interpretations; otherwise, users may misinterpret the information. The potential for misinterpretation requiring user support and contextual information was consistent with previous studies investigating public health practitioners' opinions about visualisation tools (Joyce, 2009; Koenig et al., 2011). Misinterpretation has been one of the concerns with public health data visualisations (Carroll et al., 2014), particularly when publicly accessible, due to the complicated measures and the need for some contextual information to help with interpretation. Solutions include working with domain experts to identify the contextual information relevant to charts representing complicated measures and ensuring that support is provided for new chart types to avoid misinterpretations.

Furthermore, the study findings suggest that data limitations should be communicated to prevent user distrust, and guidance should be provided to help users learn about navigation and interaction possibilities. Although missing data can influence the interpretations of health disparities (Ansari et al., 2022), the current study found that the reason for missing data was not apparent to many domain experts, and they needed to see an explanation for this and possible effects. Moreover, although data dashboards are increasingly used in non‐professional settings, such as news, this study found that interaction possibilities (even the ubiquitous filters) were not necessarily apparent to all participants. To the author's knowledge, these were new requirements that had not been identified in the previous literature. These requirements are sometimes overlooked because designers are well‐versed in different dashboards and datasets, and interaction possibilities and data limitations may seem natural. Potential solutions for adding explanations and guidance to visualisations include tooltip messages and suggested interactivity (Boy et al., 2016) to help users fully explore the affordances.

The identified requirements have several implications for health librarians and information professionals when evaluating data dashboards as trusted health information sources. First, to avoid misinterpretation, information professionals should evaluate whether the information is presented with familiar charts in simple layouts and that support is available for understanding new chart types and complicated measures on the data dashboard. Second, information professionals should consider whether contextual information is provided to help users with interpretations. Third, information professionals should evaluate the availability and understandability of explanations about data limitations on public health data dashboards to help the consumers understand the reason for these limitations and their potential impacts on the interpretations. Fourth, information professionals should evaluate whether the interaction possibilities on the public health data dashboards are clearly explained so the user can take advantage of all the provided affordances. Finally, to fit the diverging needs of different user groups, some data dashboards may use a segmentation strategy and provide different versions for their diverse user groups (Dasgupta & Kapadia, 2022). Information professionals should help users find the version of the data dashboard that best fits their needs or find other resources such as reports, narratives, and infographics if a data dashboard does not fit the users' needs.

A critique of public health data dashboards as sources of health information is that they might not be accessible to individuals with lower information and health literacy (Dasgupta & Kapadia, 2022). The current study's findings suggest that this concern can be partially addressed by ensuring that public health data dashboards meet the identified requirements. However, public health data dashboards might need time to circle among different population groups and either mature into widely used information sources or transform into better, more accessible formats (Dasgupta & Kapadia, 2022). Choosing consistent measures grounded in health literacy to present universally on all public health data dashboards can be essential to making them accessible to a broad audience (Dasgupta & Kapadia, 2022; Dixon et al., 2022). Furthermore, information professionals can improve the public's knowledge by teaching skills to find, interact with, and interpret public health data dashboards.

This study had several limitations. First, participants were recruited only from one user group (domain experts). The participants worked directly with healthcare providers and patients and could represent their needs to some extent. For example, some participants expressed concerns about using outdated terminology representing sexual minority groups that could offend some patients. However, these concerns could not wholly capture the requirements of other user groups. Future studies should investigate the requirements of other user groups to compare with and complement the current study's findings. Second, although the identified requirements were not specific to STIs, the importance of some of these requirements might be different in different settings. For example, STIs had historically been a stigmatized issue, which might have made the participants more sensitive to the issues regarding stigma and misinterpretations. The importance of this requirement for other dashboards might be different.

## CONCLUSION

Providing up‐to‐date, trusted sources of health information is a critical factor in fighting misinformation and improving health literacy in the population. However, if these sources are complex and are not designed based on their users' needs, they will not be usable and useful for their intended users. The identified requirements in this study can guide health librarians and information professionals in evaluating public health data dashboards as up‐to‐date, trusted information sources for health information consumers.

## CONFLICT OF INTEREST STATEMENT

The author declares no conflicts of interest.
